# Doctors' learning habits: CME activities among Norwegian physicians over the last decade

**DOI:** 10.1186/1472-6920-7-10

**Published:** 2007-05-08

**Authors:** Magne Nylenna, Olaf G Aasland

**Affiliations:** 1 Helsebiblioteket (Norwegian Electronic Health Library)P.O. Box7004 St Olavs plass N-0130 Oslo, Norway; 2 Department of Public Health and General Practice Norwegian University of Science and Technology N-7489 Trondheim, Norway; 3 The Research Institute of the Norwegian Medical Association PO Box 1152 Sentrum N-0107 Oslo, Norway; 4 Institute of Health Management and HealthEconomics University of Oslo PO Box 1089, Blindern N-0317 Oslo, Norway

## Abstract

**Background:**

Coping with the increasing body of medical knowledge is a main challenge to all doctors. The aim of this study was to investigate self reported reading and learning habits among Norwegian doctors and their subjective ability to keep professionally updated.

**Methods:**

A cross sectional survey among a randomised sample of Norwegian doctors was undertaken in 2004 (n = 1005, response rate 71%). A similar study with many identical questions was done in 1993 (n = 1041, response rate 71%) and a comparison of the results was made.

**Results:**

Attending courses/congresses and reading medical literature were reported to be the most important sources of professional information in 2004, just like in 1993. Less time was spent on courses/congresses in 2004 than in 1993, and more time was spent on medical reading. The internet was regarded as useful for their professional life for three out of five, mostly among the younger and least among GPs.

Two out of three doctors felt that they could obtain sufficient information for keeping updated in 2004, the same proportion as in 1993. A correlation was found between subjective coping with the information and a high level of continuing medical education (CME)-activities. The information copers had a higher level of job satisfaction than non-copers.

**Conclusion:**

Over the last decade Norwegian doctors spend less time on attending courses/congresses and more time on medical reading, while the level of self perceived coping with information has been unchanged. The changing pattern of professional updating may reflect a more general individualistic trend in society. The consistent finding of a correlation between reading and attending courses, subjective coping and job satisfaction gives good reasons for recommending a high level of CME-activities among doctors.

## Background

Being professionally updated has always been regarded as an ethical prerequisite for doctors. Over time, it has also become a formal requirement. Systematic ways of acquiring the necessary information have been developed as part of continuing medical education (CME) programmes all over the world. Doctors spend much time on reading medical literature and attending congresses and meetings to keep up with the medical knowledge.

In 1993 we made a survey of Norwegian physicians' reading and learning habits and their perceived coping with the medical information [1]. We found a high CME activity among the responders, and two out of three reported that they were able to obtain sufficient information for keeping updated. The ability to cope with the medical information was closely related to doctors' well-being and job satisfaction.

In the meantime the amount of medical knowledge has been steadily growing. The US National Library of Medicine adds every weekday between 1 500 and 3 500 new references to their bibliographic database that now contains approximately 13 million references [2]. The most significant change in information technology for decades has been introduced with the Internet. This has increased doctors' access to scientific journals and other sources of information considerably. And patients' and the public' expectations to professionals are rising. On the other hand, new methods for handling knowledge have been developed. Systematic reviews produced by explicit methods and based on all available primary research have become important tools for clinicians. The Cochrane network, established in 1993 continuously publishes systematic reviews on effects of prevention and treatment [3].

To investigate how these changes have affected doctors' learning habits and their ability to cope with the medical information, a new survey has been performed. As a follow up of our 1993 study the aim of the present study has been to answer the following questions:

• What are the doctors' most important CME activities?

• Can doctors, according to their own judgement, obtain sufficient information to keep professionally updated?

• How is self perceived coping related to CME activities and job satisfaction?

• How have doctors' information seeking behaviour and their coping with medical information changed over the last decade?

## Methods

A research programme on doctors' health and working condition has been running in Norway since 1993. The initial study was based on an extensive cross-sectional questionnaire survey of a random sample of 9266 active Norwegian doctors in 1993, (approximately 80 % of the doctor workforce at the time), conducted according to a specially designed overlapping questionnaire design [4]. The total number of respondents in 1993 was 6652 (response rate 71.8 %). A representative sub sample of 1 028 doctors completed questions about their CME-activities. In addition, a representative reference panel of Norwegian doctors was established in 1994 and supplemented in 2000, which in 2004 comprised 1 419 doctors.

Two data sets are used in this study. The first set is based on the panel respondents from 2004, and the second is a merged file with data both from the 1993 survey and from 2004. This combination was possible because some of the questions about job satisfaction, coping with new information, and time spent on CME-courses and reading medical literature, were the same inn 1993 and 2004.

The 43 formal medical specialties in Norway are collapsed into seven categories: family medicine, laboratory medicine (including pathology and radiology), internal medicine (including paediatrics and neurology), surgery (including anaesthesiology, gynaecology, and obstetrics), psychiatry, and public health. In addition there is a "no specialty" category, which mainly consists of general practitioners who are not certified specialists in family medicine. Since the 1993 data did not contain information about ongoing specialisation, the combined file has a relatively large proportion of non-specialists, consisting of both young doctors in training and older general practitioners without a formal specialty.

Our main outcome variable is dichotomous and based on the yes or no answer to the question "Can you obtain sufficient information to keep professionally updated in your daily work?".

Job satisfaction is measured by the Job satisfaction scale (JSS) based on the work by Warr et al [5]. The scale spans from 10 (low satisfaction) to 70 (high satisfaction) and has a close to normal distribution.

Both in 2004 and 1993 we asked how much time the doctors spent reading medical journals and medical textbooks, and we calculated the number of hours per week for each and their sum. The distribution of these time variables is typically lognormal.

We have used 95% confidence intervals or chi square to test significance of differences between 2004 and 1993, or among sex, age or specialty categories, and ANOVA or Mann Whitney test for comparing interval variables across groups. For multivariate analysis of the outcome variable we have used logistic regression.

## Results

A total of 1 005 doctors completed the questionnaire in 2004, a response rate of 71 % (1 005/1 419). 329 of the respondents were females (33 %) and 671 males. The mean age was 46 years (42 years for females, and 48 years for males), and 501 respondents (50 %) were under 50 years. Of the 976 with available information about specialty, 26% worked in general practice, 28 % in internal medicine, 19 % in surgical disciplines (including anaesthesiology and OBGYN), 13% in psychiatry, 9% in laboratory medicine (including radiology and pathology), and 2% in public health.

Attending professional courses and congresses and reading medical journals were reported to be the most important sources of information in 2004 just like in 1993 (table 1). Only 1 % of the respondents had not attended any professional meeting or congress during the last 12 months, compared with 2 % in 1993. 22 % had spent 1 to 5 days (13 % in 1993), 61 % had spent 6 to 15 days (59 % in 1993) and 16 % had spent more than 15 days on such activities (25 % in 1993). There was a significant trend towards fewer days spent on meetings and congresses in 2004 compared to 1993 (p < 0.001). The rank order of the importance of different CME activites was mainly unchanged over the decade (table 1).

**Table 1:** Importance of selected educational activities for professional updating shown as the proportion (%) indicating the activity to be of great or very great importance.

	2004 (N = 1005)	1993 (N= 1041)
Congresses, CME courses	82.4	79.9
Reading medical journals	70.8	65.4
Formalized meetings at workplace*	56.2	46.0
Reading textbooks etc	51.5	46.5
Informal contact with colleagues	44.6	44.5
Referral information, feedback from colleagues	33.7	38.4
Feedback from patients	30.2	34.5
Formalized supervision*	26.7	38.6
Own research	20.4	20.0
Systematic self-evaluation	18.3	21.3
Notices from health authorities	13.5	12.0
Industry ads and information	13.1	10.7
Lay media	1.0	2.1

*) Statistically significant differences due to not overlapping 95 % confidence intervals

Ranking according to importance in 2004.

Both in 1993 and 2004 the median hours per week spent on reading medical journals was two, and the time spent on medical textbooks etc. was one hour. However, the median of the sum of the two time variables increased from 3.5 hours in 1993 to 4 hours in 2004. This increase was significant among doctors in internal medicine, psychiatry and public health (p = .027, Mann Whitney non-parametric test for two independent samples).

98.5 % of the respondents had access to the internet, 73 % had access both from home and office, 15.6 % from home and 9.9 % from their office only. 63.2 % reported that the internet was fairly or very useful for their professional life. No difference was found between male and female doctors, but the internet was more important to younger than to older doctors. 71 % of doctors under 40 years reported the internet to be fairly or very useful, compared with 45.1 % among doctors over the age of 60. Important differences were also found between specialities, GPs reporting the internet to be of least importance (table 2). 31.9 % of all respondents received patient inquiries by e-mail, though only 8.5 % frequently.

**Table 2:** Usefulness of the internet for professional purposes given as the percentage of respondents reporting the internet to be fairly or very useful

	**%**
General practitioners (n = 195)	47.1
Laboratory doctors (n = 67)	71.6
Internists (n = 190)	67.9
Surgeons (n = 152)	67.8
Psychiatrists (n = 99)	60.6
Public health doctors (n = 22)	72.7
Non-specialists (n = 255)	67.0
**Total (n = 943)**	**63.2**

68.3 % of the responders reported that they can obtain sufficient information for keeping updated in their daily work. The corresponding proportion in 1993 was 67.7 %. We termed those who answered "yes" on this question "copers" and tried to characterise them in a logistic regression model. Sex, age, specialty, number of CME-course days last year, number of hours spent on reading medical literature last week, job satisfaction and general satisfaction were entered as predictor variables, and cohort (1993 or 2004) as covariates, see table 3. We found that male doctors were more likely to be copers than female doctors (OR 1.9, 95% CI 1.5 to 2.4), while age was not a significant predictor. Psychiatrists were significantly more likely to be copers than the reference group of non-specialists. Laboratory doctors were less likely to be copers than the reference group of non-specialist even though they more likely to be copers in 2004 than in 1993. Doctors with five or more CME course days last year were significantly more likely to be copers than those who had not attended courses, and there was a strong positive effect of the number of hours spent per week on medical reading. High job satisfaction was strongly associated with doctors' self-reported ability to obtain sufficient information to keep professionally updated.

**Table 3:** Factors that influence doctors' ability to obtain sufficient information to keep updated.

	B	S.E.	p	OR with 95 % CI		
	
Sex (male)	.646	.119	.000	1.91	1.51	2.41
Age (per year)	-.007	.007	.319	.99	.98	1.01

Specialty, reference: no specialty			.001			
Family medicine	.219	.255	.390	1.25	.76	2.05
Laboratory medicine	-1.201	.365	.001	.30	.15	.62
Internal medicine	-.204	.244	.403	.82	.51	1.32
Surgical disciplines	.204	.262	.434	1.23	.74	2.05
Psychiatry	.852	.390	.029	2.34	1.09	5.03
Public health	-.060	.361	.868	.942	.46	1.91

Cohort (2004)	-.283	.190	.135	.753	.52	1.09

Cohort * specialty, reference: 1993			.101			
Family medicine in 2004	.213	.331	.520	1.24	.65	2.37
Laboratory medicine in 2004	1.112	.469	.018	3.04	1.21	7.62
Internal medicine in 2004	.532	.318	.094	1.70	.91	3.178
Surgical disciplines in 2004	.631	.351	.072	1.88	.95	3.74
Psychiatry in 2004	-.273	.472	.562	.76	.30	1.92
Public health in 2004	.800	.677	.237	2.23	.59	8.39

CME days last year, reference: none			.014			

1 to 5	.536	.419	.200	1.71	.75	3.89
6 to 10	.883	.412	.032	2.42	1.08	5.42
11 to 15	.924	.416	.026	2.52	1.12	5.69
16 to 30	.967	.424	.023	2.63	1.15	6.04
more than 30	1.488	.553	.007	4.43	1.50	13.09

Hours per week spent on reading medical literature (ln)	.435	.091	.000	1.55	1.29	1.85

Job satisfaction, scale from 10 to 70	.045	.006	.000	1.05	1.03	1.06

Constant	-3.038	.528	.000	.048		

Logistic regression, N = 1797, data from 1993 and 2004

## Discussions and conclusions

The main results of the 2004 survey show striking similarities with our findings in 1993. Norwegian doctors have a fairly stable CME activity. Congresses and CME courses are still regarded the most important educational activity for Norwegian doctors, and two out of three think they can obtain sufficient information to keep professionally updated.

The main limitation of this study is that all results are based exclusively on the doctors' self reporting, their self-assessed activities and their own judgement of their ability to obtain sufficient information. As part of a comprehensive survey on doctors' health and working conditions there is, however, no reason to believe that self reported data on learning activities should be less reliable than other data collected. Perceived coping is a subjective variable and can only be monitored by self reporting. A response rate of over 70 % is acceptable and the respondents can be seen as representative for Norwegian doctors as a whole.

CME activities for doctors serve several purposes. The ultimate goal is to improve the quality of health care. It aims at updating doctors' professional knowledge and skills, and it has obvious implications on doctors' attitudes and behaviour. CME activities are closely related to patient care, and the culture of continuing education has great importance for clinical practice [6-8]. Norwegian doctors have a conservative approach to CME and they judge traditional congresses to be the most important activity for keeping up to date. More interactive educational activities, like own research and systematic self-evaluation, are still judged to be of low importance. Whether and to what extent CME activities can change doctors' professional practice is still unclear. Attending congresses and courses have little impact on improving professional practice [9,10]. It seems like interactive workshops can result in changes in practice while didactic sessions alone are unlikely to have any impact [11].

Less attendance to meetings and attending congresses over the last decade and a trend towards more individual reading might reflect more individualism in society at large. People are not only "bowling alone" as expressed by Putnam [12], but also learning alone. It may also reflect a shift towards more active and self-directed learning. Easier access to the medical literature (i.e. by the internet) facilitates individual reading. New information technology gives new opportunities [13,14].

Two out of three doctors see themselves able to obtain sufficient information to keep professionally updated. This proportion was exactly the same in 2004 as in 1993. This supports the stability and reliability of the finding. The consistent gender difference (more males than females reporting coping) is thought provoking and might be caused by basic differences in professional self-esteem between the sexes [15]. Female doctors have been shown to report less work control than their male colleagues [16]. Perhaps female doctors are more aware of their professional limitations than their male colleagues?

A high proportion of copers among psychiatrists and a low proportion among laboratory doctors (table 3) should be seen in light of the differences in knowledge production between the two specialties.

There have been important changes as regards medical information in society over the last decade. The body of medical knowledge has increased and information has become more easily available to doctors and patients alike. In general the education level of the population has increased and patients' and health authorities' demands to doctors' professional level of knowledge is higher than ever. The constant proportion of information copers among doctors in spite of all this, may reflect that the experience of keeping updated is personal and subjective and mainly based on internal factors. The correlation between CME activities like reading and attending conferences and coping with the information gives, however, reason to believe that giving priority to courses and reading is effective, even though causal relations cannot be shown in a cross sectional study.

A high level of job satisfaction is a goal for professional development. Job satisfaction seems to have a protective effect against negative consequences of work stress [17]. Participation in CME activities has been shown to be associated positively with job satisfaction and negatively with job stress [18]. The consistent finding of a strong correlation between job satisfaction and a subjective feeling of coping which again is associated with professional reading and attending CME courses, gives in itself good reasons to recommend a high degree of learning activities among doctors.

## Competing interests

The author(s) declare that they have no competing interests.

## Authors' contributions

MN and OGAa jointly designed the study, analysed the data and wrote the paper. They are both garantors.

## Pre-publication history

The pre-publication history for this paper can be accessed here:



## References

[B1] Nylenna M, Aasland OG, Falkum E (1996). Keeping professionally updated : Perceived coping and CME profiles among physicians. Journal of Continuing Education in Health Professions.

[B2] http://www.nlm.nih.gov/pubs/factsheets/medline.html.

[B3] http://www.thecochranelibrary.com.

[B4] Aasland OG, Olff M, Falkum E, Schweder T, Ursin H (1997). Health complaints and job stress in Norwegian physicians. The use of an overlapping questionnaire design. Soc Sci Med.

[B5] Warr P, Cook J, Wall T (1979). Scales for the measurement of some work attitudes and aspects of psychological well-being. J Occup Psychol.

[B6] Shannon S (2003). Educational objectives for CME programmes. Lancet.

[B7] Zeiger RF (2004). Towards continuous medical education. J Gen Intern Med.

[B8] Towle A (1998). Continuing medical education: Changes in health care and continuing medical education for the 21^st ^century. BMJ.

[B9] Davis DA, Thomson MA, Oxman AD, Haynes RB (1995). Changing physician performance. A systemtic review of the effect of continuing medical education strategies. JAMA.

[B10] Bero LA, Grilli R, Grimshaw JM, Harvey E, Oxman AD, Thomson MA (1998). Closing the gap between research and practice: an overview of systematic reviews of interventions to promote the implementation of research findings. BMJ.

[B11] O'Brien MA, Freemantle N, Oxman AD, Wolf F, Davis DA, Herrin J (2001). Continuing education meetings and workshops: effects on professional practice and health care outcomes. Cochrane Database of Systematic Reviews.

[B12] Putnam R (2000). Bowling alone.

[B13] Bergeron B (2006). Online CME options: an update. J Med Pract Manage.

[B14] Harden RM (2005). A new vision for distance learning and continuing medical education. J Contin Educ Health Prof.

[B15] Riska E (2001). Towards gender balance: but will women physicians have an impact on medicine?. Soc Sci Med.

[B16] McMurray JE, Linzer M, Konrad TR, Douglas J, Shugerman R, Nelson K (2000). The work lives of women physicians. J Gen Intern Med.

[B17] Visser MRM, Smets EMA, Oort FJ, de Haes HCJM (2003). Stress, satisfaction and burnout among Dutch medical specialists. Can Med Assn J.

[B18] Kushnis T, Cohen AH, Kitai E (2000). Continuing medical education and primary physicians' job stress, burnout and dissatisfaction. Med Educ.

